# Us3 Kinase Encoded by Herpes Simplex Virus 1 Mediates Downregulation of Cell Surface Major Histocompatibility Complex Class I and Evasion of CD8^+^ T Cells

**DOI:** 10.1371/journal.pone.0072050

**Published:** 2013-08-12

**Authors:** Takahiko Imai, Naoto Koyanagi, Ryo Ogawa, Keiko Shindo, Tadahiro Suenaga, Ayuko Sato, Jun Arii, Akihisa Kato, Hiroshi Kiyono, Hisashi Arase, Yasushi Kawaguchi

**Affiliations:** 1 Division of Molecular Virology, Department of Microbiology and Immunology, the Institute of Medical Science, the University of Tokyo, Tokyo, Japan; 2 Division of Viral Infection, Department of Infectious Disease Control, International Research Center for Infectious Diseases, the Institute of Medical Science, the University of Tokyo, Tokyo, Japan; 3 Nippon Institute for Biological Science, Ome, Tokyo, Japan; 4 Division of Mucosal Immunology, Department of Microbiology and Immunology, the Institute of Medical Science, the University of Tokyo, Tokyo, Japan; 5 Core Research for Evolutional Science and Technology, Japan Science and Technology Agency, Saitama, Japan; 6 Department of Immunochemistry, Research Institute for Microbial Diseases, Osaka University, Suita, Osaka, Japan; 7 World Premier International Research Center Immunology Frontier Research Center, Osaka University, Suita, Osaka, Japan; Lisbon University, Portugal

## Abstract

Detection and elimination of virus-infected cells by CD8^+^ cytotoxic T lymphocytes (CTLs) depends on recognition of virus-derived peptides presented by major histocompatibility complex class I (MHC-I) molecules on the surface of infected cells. In the present study, we showed that inactivation of the activity of viral kinase Us3 encoded by herpes simplex virus 1 (HSV-1), the etiologic agent of several human diseases and a member of the *alphaherpesvirinae*, significantly increased cell surface expression of MHC-I, thereby augmenting CTL recognition of infected cells in vitro. Overexpression of Us3 by itself had no effect on cell surface expression of MHC-I and Us3 was not able to phosphorylate MHC-I in vitro, suggesting that Us3 indirectly downregulated cell surface expression of MHC-I in infected cells. We also showed that inactivation of Us3 kinase activity induced significantly more HSV-1-specific CD8^+^ T cells in mice. Interestingly, depletion of CD8^+^ T cells in mice significantly increased replication of a recombinant virus encoding a kinase-dead mutant of Us3, but had no effect on replication of a recombinant virus in which the kinase-dead mutation was repaired. These results indicated that Us3 kinase activity is required for efficient downregulation of cell surface expression of MHC-I and mediates evasion of HSV-1-specific CD8^+^ T cells. Our results also raised the possibility that evasion of HSV-1-specific CD8^+^ T cells by HSV-1 Us3-mediated inhibition of MHC-I antigen presentation might in part contribute to viral replication in vivo.

## Introduction

Herpes simplex virus-1 (HSV-1) is the member of the *Alphaherpesvirinae*, the neurotropic subfamily of herpesviruses [1]. Like other herpesviruses, HSV-1 causes a life-long infection cycling between lytic and latent phases in the natural human host [1]. This HSV-1 life-cycle repeatedly primes the host immune system, thereby increasing the potential for the host to eradicate the virus. To overcome this situation, HSV-1 has had to evolve mechanisms to evade immune detection and clearance [2,3].

CD8^+^ cytotoxic T lymphocytes (CTLs) play an important role in the clearance of herpesvirus-infected cells [2,3]. CD8^+^ CTLs recognize viral antigens presented as peptides bound to major histocompatibility complex class I (MHC-I) molecules, which are assembled in the endoplasmic reticulum (ER), at the surface of infected cells [4]. Therefore, the MHC-I antigen presentation pathway seems to be a prime target for herpesviruses to attack to evade the host immune system. In fact, numerous reports have shown that various herpesvirus proteins have evolved to inhibit MHC-I antigen presentation in vitro by a variety of mechanisms [2,3]. However, the data that these in vitro mechanisms affect viral replication and pathogenesis in vivo is limited to some non-human, animal herpesviruses in the *Betaherpesvirinae* or *Gammaherpesvirinae* [5–7].

HSV has been reported to encode two viral proteins, ICP47 and virion host shutoff protein (vhs), that inhibit antigen presentation on MHC-I to CD8^+^ CTLs in vitro [8,9]. ICP47 binds to the transporter associated with antigen processing (TAP), which translocates antigen peptides derived from proteosomal degradation of viral proteins into the endoplasmic reticulum (ER) [4], where the antigen peptides are loaded onto newly synthesized MHC-I [4]. ICP47 acts as a high-affinity competitor for peptide binding to TAP, thereby inhibiting MHC-I antigen presentation on the surface of HSV-infected cells [10,11]. While HSV ICP47 efficiently inhibits MHC-I antigen presentation in human cells [8], inhibition of antigen presentation in murine cells is only marginally effective, due to about a 100-fold decrease in ICP47 binding to murine TAP compared to human TAP [11,12]. Consistent with these data, ICP47 protects HSV-infected human ﬁbroblasts from destruction by CD8^+^ CTLs, while mouse ﬁbroblasts are not protected [13]. These ICP47 properties make it difficult to address the importance of ICP47-mediated inhibition of MHC-I presentation of HSV antigens in murine models, which have been extensively used to study the pathogenesis and immunological control of HSV infection. In contrast, despite the limited ability of ICP47 to inhibit mouse TAP, a role for ICP47 in evasion of CD8^+^ T cell-mediated immunity in mice was suggested by a study showing that CD8^+^ CTLs were able to protect mice from an HSV mutant lacking ICP47 but not from wild-type virus [14]. Thus, the mechanism by which ICP47 acts in evasion of CD8^+^ CTLs in mice remains uncertain at present. vhs, another HSV protein involved in evasion of CD8^+^ CTLs by inhibition of MHC-I antigen presentation, is an mRNA-specific RNase that triggers rapid shutoff of host cell protein synthesis [15] and inhibits synthesis of MHC-I in HSV-infected cells [9]. HSV-2 vhs has been reported to help infected cells become resistant to lysis by CD8^+^ CTLs in vitro [9]. However, vhs appears not to play a role in evasion of CD8^+^ CTLs in vivo, based on the observation that a vhs-null mutation in HSV-2 attenuated viral replication and pathogenesis in SCID mice to levels similar to those in normal mice [16].

In some alphaherpesviruses [e.g., bovine herpesvirus 1 (BHV-1), pseudorabies virus (PRV), equine herpesvirus 1 and 4 (EHV-1 and EHV-4) and Marek’s disease virus], UL49.5 homologs have been reported to inhibit MHC-I antigen presentation by affecting the function of TAP [17–19]. However, in other alphaherpesviruses [e.g., HSV and varicella zoster virus (VZV)], UL49.5 homologs are not involved in inhibition of MHC-I antigen presentation, suggesting that the role of conserved alphaherpesvirus gene products in MHC-I antigen presentation may vary. VZV ORF66, a serine/threonine protein kinase, has also been reported to down-regulate cell surface expression of MHC-I by blocking transport of mature MHC-I through the cis/medial-Golgi complex [20]. In addition, PRV Us3, a homolog of VZV ORF66, was shown to be required, but not sufficient, for downregulation of cell surface expression of MHC-I [21]. Moreover, HSV-1 Us3 has been reported to collaborate with viral envelope glycoprotein B (gB) to downregulate cell surface expression of MHC-I-like antigen-presenting molecule CD1d to potently inhibit its recognition by CD1d-restricted natural killer T cells [22]. UL56 homologs in EHV-1 and EHV-4 were also recently reported to down-regulate cell surface expression of MHC-I [23,24]. However, it remains to be determined whether UL49.5, ORF66, Us3 and UL56 proteins protect virus-infected cells from destruction by CD8^+^ CTLs in vitro and whether their potential immune evasion functions contribute to viral replication in vivo.

In the present study, we showed that the activity of HSV-1 Us3 was required for efficient inhibition of MHC-I antigen presentation to prevent CTL recognition of infected cells in vitro and for downregulation of induction of HSV-1-specific CD8^+^ T cells in mice. Us3 appreared to indirectly downregulate MHC-I since Us3 was not sufficient for MHC-I downregulation and Us3 was not able to phosphorylate MHC-I in vitro. Depletion of CD8^+^ T cells in mice significantly increased replication of a recombinant virus encoding a kinase-dead mutant of Us3, but had no effect on replication of a recombinant virus in which the kinase-dead mutation was repaired. Our results raised the possibility that inhibition of MHC-I antigen presentation mediated by HSV-1 Us3 kinase activity might in part contribute to viral replication in vivo.

## Materials and Methods

### Cells and viruses

**Figure 1:**
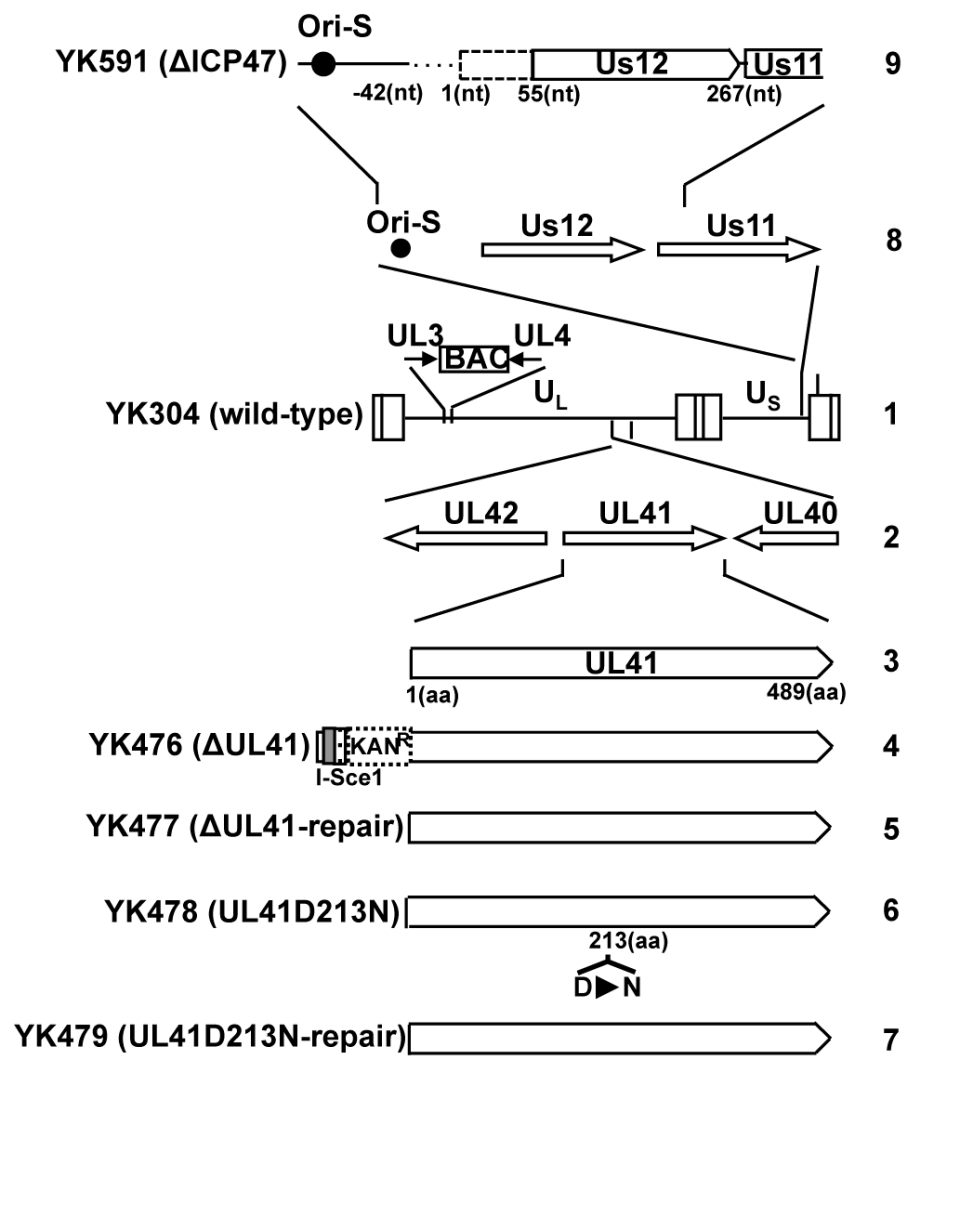
Schematic diagram of the genome structures of wild type YK304 and the relevant domains of the recombinant viruses used in this study. Line 1, YK304 genome carrying a bacmid (BAC) in the intergenic region between UL3 and UL4. Line 2, domains encoding the UL40 to UL42 open reading frames. Line 3, UL41 gene encoding vhs. Lines 4-7, recombinant viruses with mutations in the UL41 gene. Line 8, domains encoding the Us11 to Us12 open reading frames and the viral replication origin S (Ori-S). Line 9, recombinant virus with mutation in the Us12 gene encoding ICP47.

Vero, 293T and B6MEFs, an immortalized mouse embryonic fibroblast (MEF) cell line derived from wild-type C57BL/6J mice, were described previously [25,26]. MRC-5 cells, human normal embryonic lung fibroblasts, were obtained from the Riken BioResource Center. MHC-I^-/-^MEFs, an immortalized MEF cell line derived from C57BL/6J mice lacking the gene encoding MHC-I (a gift from Dr. A.B. Hill), and HSV-2.3.2E2 cells, a *lacZ*-inducible CD8^+^ T cell hybridoma that recognizes HSV-1 gB_498–505_ (a gift from Dr. F.R. Carbone), are described elsewhere [27]. HSV-1 wild-type strain HSV-1(F), recombinant virus YK511 encoding an enzymatically inactive Us3 mutant in which lysine at Us3 residue 220 was replaced with methionine (Us3-K220M), recombinant virus YK513 in which the Us3 K220M mutation in YK511 was repaired (Us3-repair) and reombinant virus YK476 (ΔUL41) in which the UL41 gene was disrupted by the insertion of a foreign gene casette carrying a stop codon just downstream of the UL41 start codon, an I-SceI site, a kanamycin resistance gene, and 60 bp of a sequence upstream of the second codon of the UL41 gene (Fig. 1) were described previously [28]. Recombinant virus YK477 (ΔUL41-repair) in which the foreign gene cassette inserted into the UL41 locus of YK477 was excised (Fig. 1) was generated as described previously [28] except using *E. coli* containing the YK477 genome. Recombinant virus YK478 encoding UL41 with an aspartic acid substitution for asparagine at amino acid 213 (UL41D213N) (Fig. 1) was generated as described previously [29] except using primers 5’- CCTCTATCACACCAACACGGTCGCGTACGTGTACACCACGAACACTGACCTCCTGTTGATGAGGATGACGACGATAAGTAGGG-3’ and 5’- TCCAACACAATATCGCAGCCCATCAACAGGAGGTCAGTGTTCGTGGTGTACACGTACGCGACAACCAATTAACCAATTCTGATTAG-3’. Recombinant virus YK479, in which the UL41D213N mutation in YK478 (Fig. 1) was repaired, was genenerated as described previously [29] except with the primers 5’- CCTCTATCACACCAACACGGTCGCGTACGTGTACACCACGGACACTGACCTCCTGTTGATGAGGATGACGACGATAAGTAGGG-3’ and 5’- TCCAACACAATATCGCAGCCCATCAACAGGAGGTCAGTGTCCGTGGTGTACACGTACGCGACAACCAATTAACCAATTCTGATTAG-3’. Recombinant virus YK591 with a 100 bp deletion (42 bp upstream to 55 bp downstream of the start codon) in ICP47 (ΔICP47) (Fig. 1) was constructed by the two-step Red-mediated mutagenesis procedure as described previously [29], except using primers 5’-TTGCGTGGACCGCTTCCTGCTCGTCGGGGCGACCGGCGGCGGACGTACGCCGACGTACGCAGGATGACGACGATAAGTAGGG-3’ and 5’-CCCCTTTTATTGATCTCATCGCGTACGTCGGCGTACGTCCGCCGCCGGTCGCCCCGACGACAACCAATTAACCAATTCTGATTAG-3’. This deletion in ICP47 was reported to eliminate ICP47 expression and have no effect on expression of the neighboring Us11 gene [14].

### Analysis of cell surface expression of MHC-I

Cell surface and total expression of human and mouse MHC-I in infected cells was determined as described previously [30,31], except that FITC-conjugated anti-human-HLA-ABC (G46-2.6; Becton Dickinson), anti-mouse-H-2Kb (AF6-88.5; BioLegend) and anti-mouse-H-2Db (28-14-8; eBioscinece) antibodies were used. Cell surface expression of human MHC-I in 293T cells co-transfected with pEGFP-C1 (clontech) in combination with pFLAG-CMV-2 or pFLAG-Us3 was analyzed as described above, except that EGFP-positive cells were gated, and anti-human HLA-ABC antibody (G46-2.6 Becton Dickinson) and Alexa Fluor 680 Goat Anti-mouse IgG F(ab’)2 fragment (Invitrogen) were used. pFLAG-Us3 was constructed by cloning the entire coding sequences of Us3 and Us3 carrying K220M mutation amplified from pBS-Us3 [32] into pFlag-CMV-2 (Sigma). 293T cells transfected with pFLAG-Us3 expressed Us3 efficiently (data not shown).

**In vitro kinase assay**. pMAL-gB-P2 and pMAL-gB-P2-T887A were described previously [31]. To generate a fusion protein of maltose binding protein (MBP) and part of MHC-I, pMAL-MHC-I was constructed by amplifying the domain encoding MHC-I codons 211-365 by PCR from a HeLa cDNA library (BioChain) and cloning the DNA fragments into pMAL-c (New England BioLabs) in frame with MBP. To generate a fusion protein of MBP fused to part of MHC-I in which the serines at MHC-I residues 336 and 337, which are potential PKA phosphorylation sites, were replaced with alanines (MHC-I-SS336/337AA), pMAL-MHC-I was mutated to generate pMAL-MHC-I-SS336/337AA as described previously [31]. MBP fusion proteins MBP-gB, MBP-gB-T887A, MBP-MHC-I and MBP-MHC-I-SS336/337AA were expressed in *E. coli* that had been transformed with pMAL-gB-P2, pMAL-gB-P2-T887A, pMAL-MHC-I and pMAL-MHC-I-SS336/337AA, respectively, and purified as described previously [31]. The purified MBP fusion proteins were captured on amylose beads (New England BioLabs) and used as substrates for in vitro kinase assays, as described previously [31], with glutathione S-transferase (GST)-Us3 purified as described previously [32] and protein kinase A (PKA) purchased from New England BioLabs.

### Detection of antigen presentation in infected cells

B6MEF and MHC-I^-/-^MEF cells were grown in 24-well plates and infected with each of the indicated viruses at an MOI of 1. At 12 h post-infection, 5 x 10^4^ HSV-2.3.2E2 cells were added to each well and incubation was continued for an additional 12 h. LacZ expression was then determined as described previously [5].

### Quantitative RT-PCR

Total RNA was isolated from infected cells with a High Pure RNA Isolation Kit (Roche) and cDNA was synthesized from the isolated RNA with a Transcriptor First Strand cDNA Synthesis Kit (Roche) according to the manufacturer’s instructions. The amount of cDNA of specific genes was quantitated using the Universal ProbeLibrary (Roche) with TaqMan Master (Roche) and the LightCycler 1.5 System (Roche) according to the manufacturer’s instructions. Gene-specific primers and universal probes were designed using ProbeFinder software (Roche). The primer and probe sequences for human β-actin were 5’-CCAACCGCGAGAAGATGA-3’, 5’-CCAGAGGCGTACAGGGATAG-3’ and Universal ProbeLibrary probe 64; for mouse β-actin were 5’-GGAGGGGGTTGAGGTGTT-3’, 5’-GTGTGCACTTTTATTGGTCTCAA-3’ and Universal ProbeLibrary probe 71 and for 18S rRNA were 5’-GCAATTATTCCCCATGAACG-3’, 5’-GGGACTTAATCAACGCAAGC-3’ and ProbeLibrary Probe 48. The amount of β-actin expression was normalized to the amount of 18S rRNA expression. The relative amount of each gene expression was calculated using the comparative CT (2^-ΔΔCt^) method [33].

### Preparation of NK cells and analysis of γ-IFN production

NK cells were purified and cultured as described previously [34]. For analysis of γ-IFN production, 2x10^5^ NK cells were co-cultured with 2 x 10^5^ B6MEFs infected with each of the indicated viruses for 24 h. Enzyme-linked immunosorbent assays (ELISA) were performed using Mouse IFN gamma ELISA Ready-SET-Go! (eBioscience) according to the manufacturer’s instructions.

### Animal studies

Three or two six-week-old female C57BL/6J mice were mock-infected or infected with 1 x 10^6^ PFU of the indicated virus/footpad. At 4 d post infection, spleen and popliteal lymph node white blood cells from infected mice sacrified by cervical dislocation were stained with MHC-I tetramer for the HSV-1 gB epitope (gB_498-505_) (MBL). Cells were then stained with FITC-conjugated anti-CD8α (eBioH35-17.2; eBioscience) and APC-anti-CD3ε (145-2C11; eBioscience) antibodies as described previously [30] and analyzed with a FACSCalibur with Cell Quest software (Becton Dickinson). The results were based on 9 experiments with HSV-1(F), YK511 (Us3K220M) and YK513 (Us3K220M-repair) and 5 experiments with YK591 (ΔICP47), and expressed as the percentage of gB-specific MHC-I tetramer positive cells that stained positive for CD8α. For depletion of CD8^+^ T or NK cells in mice, 200 µg anti-CD8α (53.6.72 obtained from ATCC) or anti-NK1.1 (PK136 obtained from ATCC) antibody, respectively, was administered to 6-week-old female C57BL/6J mice by intraperitoneal injection 2 d before HSV-1 infection. Administration of these antibodies into mice routinely resulted in >95% depletion of CD8^+^ and NK1.1^+^ cells in lymph node and spleen (Fig. S1). The depleted condition was maintained by repeated injections of monoclonal antibody at 3 d intervals. Three mice depleted of CD8^+^ T or NK cells were infected with 1 x 10^6^ PFU YK511 (Us3-K220M) or YK513 (Us3-Repair)/footpad. At 1 and 4 d post-infection, mice were sacrificed by cervical dislocation and footpads were harvested, homogenized and sonicated. Viral titers in the footpads were determined by standard plaque assays on Vero cells. The results were based on two experiments. All animal experiments were carried out in accordance with the Guidelines for Proper Conduct of Animal Experiments, Science Council of Japan. The protocol was approved by the Institutional Animal Care and Use Committee (IACUC) of the Institute of Medical Science, The University of Tokyo (IACUC protocol approval number: 19-26).

## Results

### Effect of HSV-1 Us3 kinase activity on cell surface expression of MHC-I in infected human and murine cells

**Figure 2:**
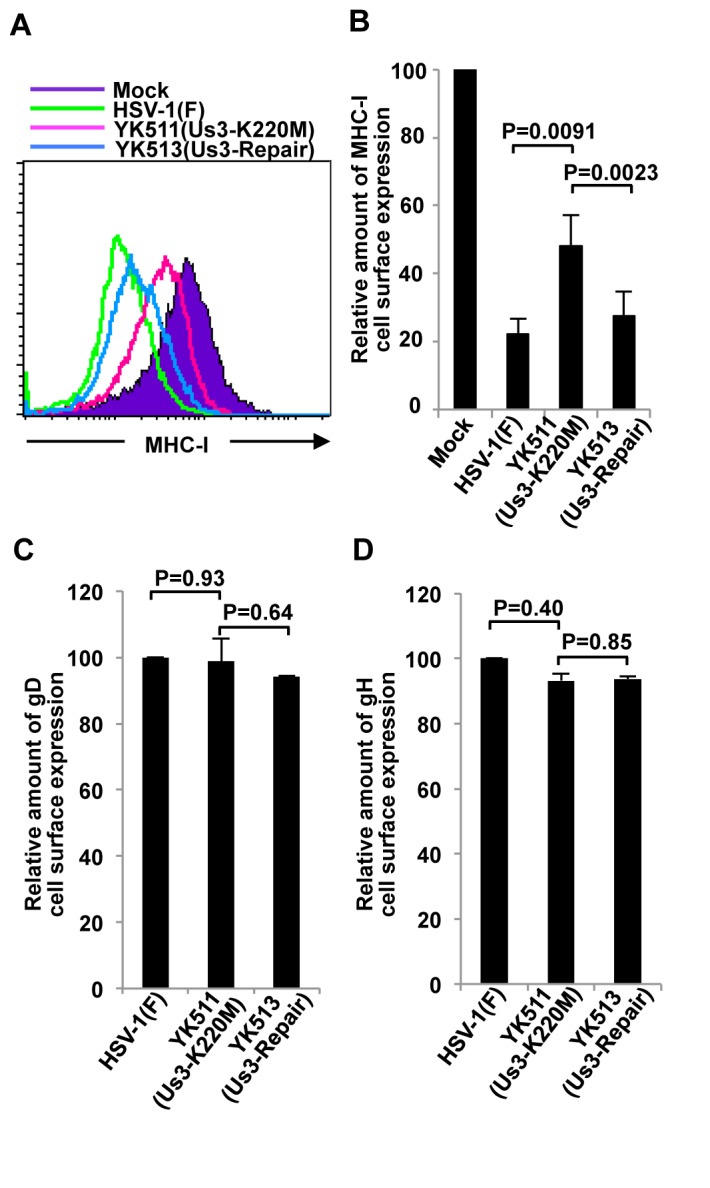
Effect of Us3 kinase activity on cell surface expression of MHC-I, gD and gH in HSV-1-infected MRC-5 cells. (A) Cell surface expression of MHC-I in human MRC-5 cells mock-infected (filled purple histogram) or infected with wild-type HSV-1(F) (green line), YK511 (Us3-K220M) (pink line) or YK513 (Us3-repair) (blue line) at an MOI of 3 for 18 h and analyzed by flow cytometry. The data are representative of five independent experiments. (B) Quantitation of cell surface expression of MHC-I in infected MRC-5 cells. The relative mean fluorescence intensity (MFI) for MHC-I expression on the surface of cells infected with the indicated virus is shown as the fluorescence intensity of virus-infected cells relative to that of mock-infected cells. Each data point is the mean ± standard error of five independent experiments. (C and D) Surface expression of gD (C) and gH (D) in MRC-5 cells infected with HSV-1(F), YK511 (Us3-K220M) or YK513 (Us3-repair) at an MOI of 3 for 18 h and analyzed and quantitated as described in Figure 2B. Each data point is the mean ± standard error of triplicate samples, and is representative of three independent experiments.

**Figure 3:**
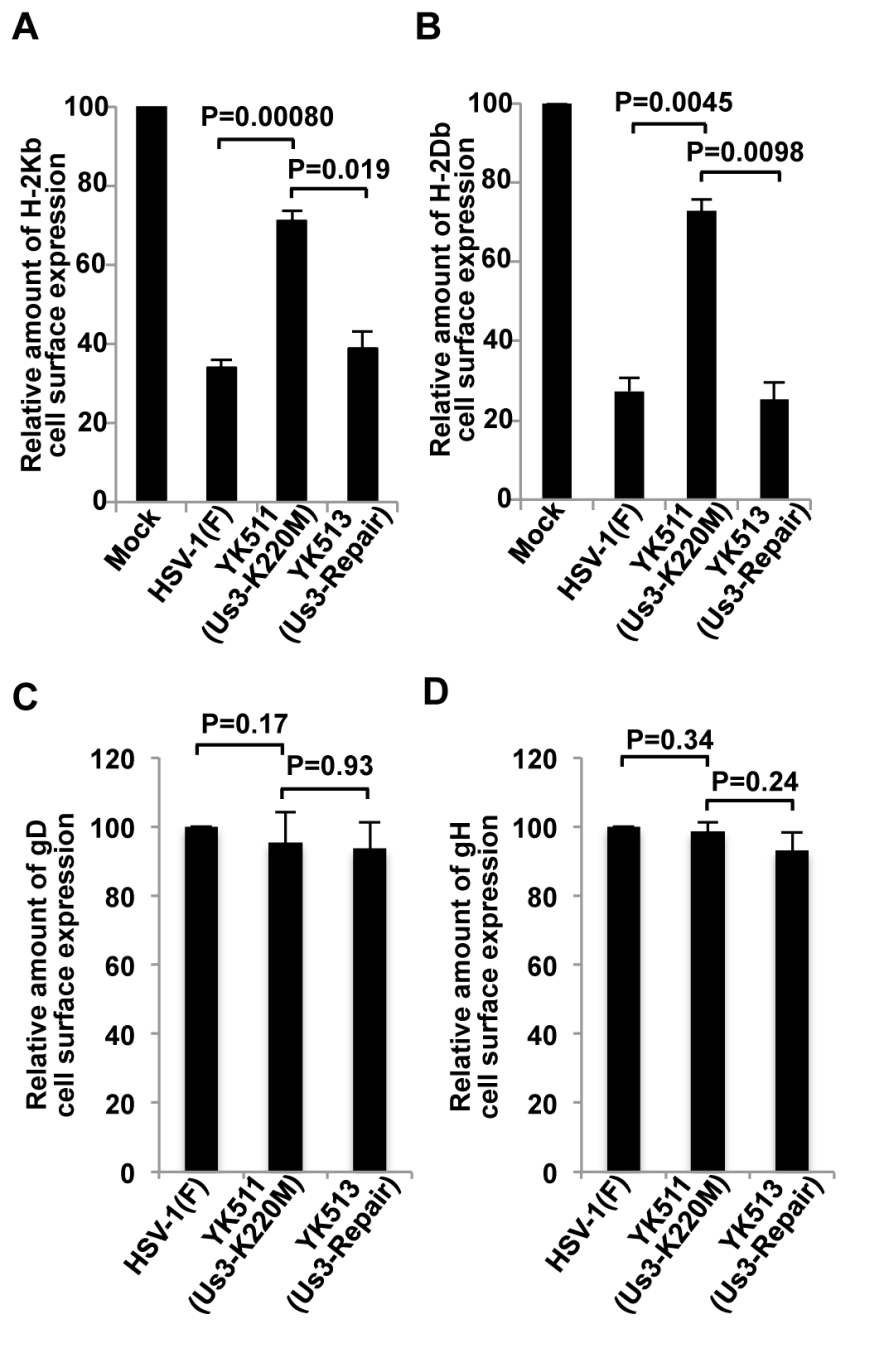
Effect of Us3 kinase activity on cell surface expression of MHC-I (H-2Kb and H-2Db), gD and gH in HSV-1-infected MEFs from C57BL/6J mice (B6MEFs). (A and B) Surface expression of H-2Kb (A) and H-2Db (B) in B6MEFs mock-infected or infected with HSV-1(F), YK511 (Us3-K220M) or YK513 (Us3-repair) at an MOI of 3 for 18 h and analyzed and quantitated as described in Figure 2B. Each data point is the mean ± standard error of three independent experiments. (C and D) Surface expression of gD (C) and gH (D) in B6MEFs infected with HSV-1(F), YK511 (Us3-K220M) or YK513 (Us3-repair) at an MOI of 3 for 18 h and analyzed and quantitated as described in Figure 2B. Each data point is the mean ± standard error of triplicate samples, and is representative of three independent experiments.

We previously reported that HSV-1 Us3 directly phosphorylates HSV-1 envelope glycoprotein B (gB) and down-regulates its cell surface expression in infected cells by promoting gB endocytosis [30,31]. Moreover, Us3 homologs in VZV and PRV down-regulate cell surface expression of MHC-I in infected cells, as described above [20] and reported by Deruelle [21]. These observations prompted us to examine whether MHC-I is also a target of HSV-1 Us3 in the regulation of its cell surface expression in infected cells. As shown in Figure 2A and B, wild-type HSV-1(F) infection of MRC-5 cells, human primary lung fibroblasts, resulted in significant down-regulation of MHC-I cell surface expression as reported previously [35]. In contrast, MHC-I cell surface expression increased significantly in MRC-5 cells infected with YK511 (Us3K220M), a Us3 kinase-dead mutant virus, compared to MRC-5 cells infected with wild-type HSV-1(F) or YK513 (Us3-repair), a recombinant virus in which the kinase-dead mutation in Us3 was repaired. However, the total accumulation of MHC-I protein in YK511-infected cells was similar to that in cells infected with HSV-1(F) or YK513 (Us3-repair) as described below (Fig. S5B). Furthermore, cell surface expression of gD and gH in MRC-5 cells infected with YK511 (Us3K220M) was similar to that in MRC-5 cells infected with wild-type HSV-1(F) (Fig. 2C and D). Similar results were also obtained for cell surface expression of H-2Kb and H-2Db in B6MEFs, a mouse cell line (Fig. 3 and Fig. S6B and D). These results indicated that Us3 kinase activity was required for efficient down-regulation of MHC-I cell surface expression in both human and murine cells infected with HSV-1.

### Effect of HSV-1 Us3 in the absence of any other viral proteins on cell surface expression of MHC-I and on phosphorylation of MHC-I in vitro

To investigate whether Us3 by itself directly acts on MHC-1 or indirectly via other Us3-dependent viral and/or cellular protein(s), we performed two series of experiments. In the first series of experiments, 293T cells were trasfected with a plasmid expressing wild-type Us3 or an empty expression plasmid and cell surface MHC-I was then analyzed. As shown in Figure S2A, expression of Us3 had little effect on cell surface expression of MHC-I. In the second series of experiments, we genenrated and purified a chmeric protein consisting MBP fused to peptides encoded by MHC-I codons 211 to 365 (MBP-MHC-I) which included the entire cytoplasmic domain of MHC-I. We also generated a mutated version of MBP-MHC-I (MBP-MHC-I-SS336/337AA) in which alanines were substituted for serines at positions 336 and 337, which has been reported to be phosphorylated by protein kinase A (PKA) [36]. These MBP fusion proteins were used as substrates for in vitro kinase assays with purified GST-Us3 and PKA. MBP-gB and a mutated version of MBP-gB (MBP-gB-T887A) in which alanine was substituted for serine at position 887 which were shown to phosphorylated by GST-Us3 [31] were also used in these assays. As shown in Figure S2B, GST-Us3 did not phosphorylated MBP-MHC-I. In contrast, GST-Us3 and PKA phosphorylated MBP-gB and MBP-MHC-I, respectively, but not MBP-gB-T887A and MBP-MHC-I-SS336/337AA as reported previously [31,36] (Fig. S2B). Taken together, these results suggested that Us3 indirectly down-regulated cell surface expression of MHC-I.

### Effect of HSV-1 Us3 kinase activity on HSV-1-specific antigen presentation in infected cells

**Figure 4:**
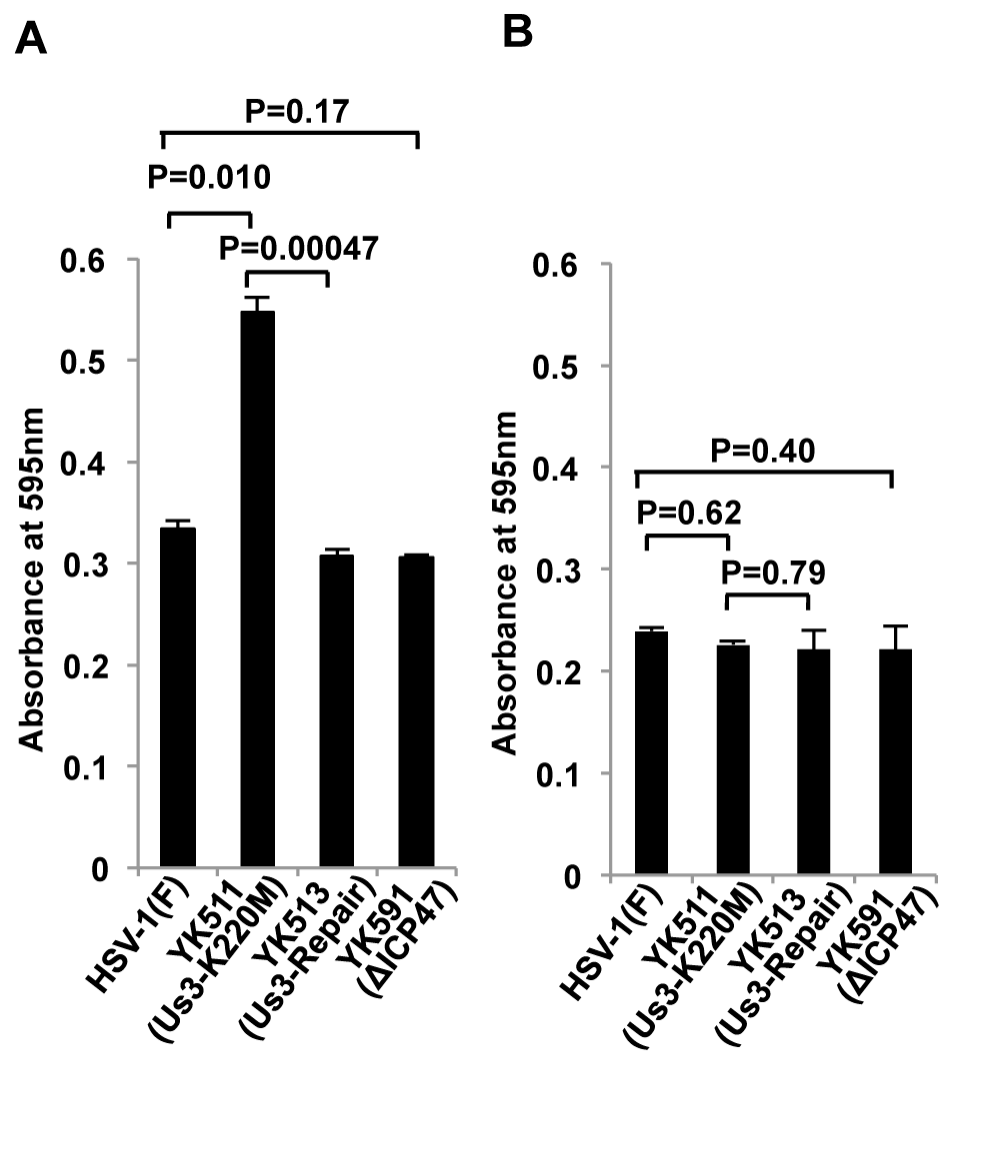
Effect of Us3 kinase activity on HSV-1-specific antigen presentation. B6MEFs (A) and MHC-I-/-MEFs (B) were infected with HSV-1(F), YK511 (Us3-K220M), YK513 (Us3-Repair) or YK591 (ΔICP47) at an MOI of 1 for 12 h and then co-cultured for an additional 12 h with *lacZ*-inducible CTL hybridoma cells recognizing HSV-1 gB (HSV-2.3.2E2), followed by β-galactosidase assays. Each data point is the mean ± standard error of triplicate samples, and is representative of three independent experiments.

Inhibition of HSV-1-specific antigen presentation mediated by Us3 kinase in infected cells was analyzed using a CTL hybridoma clone that produced β-galactosidase in response to the immunodominant gB_498-505_ epitope of HSV-1 [27]. As shown in Figure 4A, the response of the HSV-1-specific CTL hybridoma clone to B6MEFs infected with YK511 (Us3-K220M) was significantly greater than to B6MEFs infected with wild-type HSV-1(F) or YK513 (Us3-repair). This difference in the HSV-1-specific CTL hybridoma clone response was not observed in infected MHC-I^-/-^MEFs (Fig. 4B). In agreement with previous reports that ICP47 inhibits MHC-I antigen presentation poorly in mouse cells [11,12], the response of the HSV-1 specific CTL hybridoma clone to B6MEFs infected with the ICP47-null mutant virus YK591 (ΔICP47) was similar to the response of the clone to B6MEFs infected with wild-type HSV-1(F). These results indicated that Us3 kinase activity mediated the inhibition of MHC-I-restricted, HSV-1-specific antigen presentation in cultured infected cells.

### Charactirization of recombinant viruses YK511 (Us3K220M) and YK478 (UL41D213N) and their repaired viruses in MRC-5 and B6MEF cells

Ryckman and Roller previously reported that the kinase-dead mutation in Us3 impaired viral replication in HEp-2 cells but not in Vero cells [37]. To examine whether the kinase-dead mutation (K220M mutation in Us3) also impaired viral replication in MRC-5 and B6MEF cells, we analyzed viral growth of wild-type HSV-1(F), YK511 (Us3K220M) and YK513 (Us3K220M-repair) in MRC-5 and B6MEF cells infected at an MOI of 3 or 0.01. As shown in Figure S3, the progeny virus yield of YK511 (Us3K220M) in MRC-5 and B6MEF cells at both MOIs was reduced compared to wild-type HSV-1(F) and YK513 (Us3-repair) progeny virus yields.

To eliminate the possibility that the increase in MHC-I cell surface expression and MHC-I-restricted, HSV-1-specific antigen presentation in MRC-5 and B6MEF cells infected with YK511 (Us3K220M) resulted from the reduced growth of the mutant virus in these cells, we also characterized recombinant virus YK478 (UL41-D213N) carrying the D213N mutation in UL41 and its repaired virus YK479 (UL41-D213N-repair) in MRC-5 and B6MEF cells. The growth kinetics of YK478 (UL41D213N) in MRC-5 and B6MEF cells at MOIs of 3 and 0.01 was almost identical to that of YK511 (Us3K220M) (Fig. S3). The D213N mutation in UL41 has been reported to abolish vhs RNase activity in transfection experiments [38]. In agreement with this report, the level of cellular β-actin mRNA was significantly up-regulated in MRC-5 and B6MEF cells infected with YK478 (UL41-D213N) to a level similar to that in cells infected with the UL41 null mutant virus YK476 (ΔUL41), compared to the level of cellular β-actin mRNA in cells infected with wild-type HSV-1(F) and YK479 (UL41D213N-Repair) (Fig. S4), confirming that the D213N mutation in UL41 abolished vhs RNase activity in infected cells. Furthermore, we noted that the level of β-actin mRNA in MRC-5 and B6MEF cells infected with YK511 (Us3K220M) was similar to that in cells infected with wild-type HSV-1(F) (Fig. S4), indicating that the kinase-dead mutation in Us3 had no effect on vhs function in these infected cells.

We then examined total and cell surface expression of MHC-I and MHC-I-restricted, HSV-1-specific antigen presentation in MRC-5 and B6MEF cells infected with wild-type HSV-1(F), YK478 (UL41-D213N) and YK479 (UL41D213N-repair). As shown in Figure S5A, MHC-I cell surface expression in MRC-5 cells infected with YK478 (UL41D213N) was similar to that of MRC-5 cells infected with wild-type HSV-1(F) or YK479 (UL41D213N-repair). In agreement with a previous report [38], the total accumulation of MHC-I protein was significantly increased in MRC-5 cells infected with YK478 (UL41D213N) compared to MRC-5 cells infected with wild-type HSV-1(F) or YK479 (UL41D213N-repair) (Fig. S5B). Similar results were also obtained for cell surface expression of H-2Kb and H-2Db in B6MEFs (Fig. S6). Furthermore, the response of an HSV-1 specific CTL hybridoma clone to B6MEFs infected with YK478 (UL41-D213N) was similar to the response of the clone to B6MEFs infected with wild-type HSV-1(F) or YK479 (UL41D213N-repair) (Fig. S7). Thus, inactivation of vhs RNase activity in infected MRC-5 and B6MEF cells decreased viral growth to a level almost identical to that produced by inactivation of Us3 kinase activity. However, unlike inactivation of Us3 kinase activity, inactivation of vhs RNase activity in MRC-5 and B6MEF cells had no effect on MHC-I cell surface expression or on inhibition of MHC-I-restricted, HSV-1-specific antigen presentation. These results indicated that both vhs and Us3 activities were required for efficient viral replication in MRC-5 and B6MEF cells, but vhs played no role in down-regulation of MHC-I cell surface expression in infected MRC-5 and B6MEF cells or in inhibition of MHC-I-restricted, HSV-1-specific antigen presentation in infected B6MEFs.

### Effect of HSV-1 Us3 kinase activity on susceptibility of infected cells to NK cell recognition

**Figure 5:**
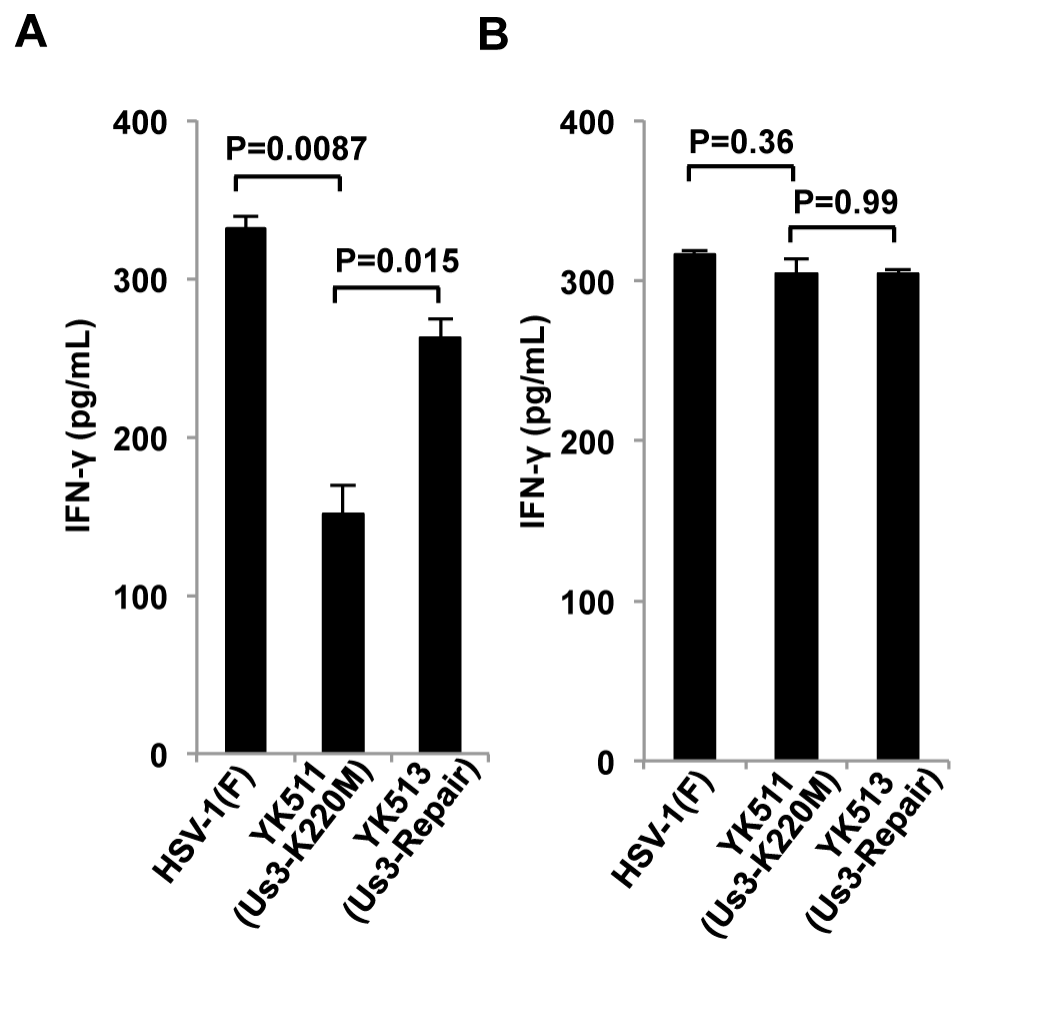
Effect of Us3 kinase activity on susceptibility of HSV-1-infected cells to NK cell recognition. B6MEFs (A) and MHC-I^-/-^MEFs (B) were infected with HSV-1(F), YK511 (Us3-K220M), or YK513 (Us3-Repair) at an MOI of 1 for 12 h and then co-cultured with NK cells isolated from C57BL/6J mouse splenocytes for an additional 24 h. IFN-γ in the co-culture cell supernatants was quantified by ELISA. Each data point is the mean ± standard error of triplicate samples, and is representative of three independent experiments.

MHC-I molecules are ligands for inhibitory receptors on NK cells [39]. Therefore, HSV-1 Us3-mediated inhibition of MHC-I cell surface expression in infected cells was proposed to increase recognition of cells infected with wild-type HSV-1 by NK cells more than cells infected with YK511 (Us3K220M). Consistent with this proposal, the amount of γ-interferon (γ-IFN), which is induced upon NK cell recognition of target cells [39], in the supernatants of IL-2-expanded NK cells co-cultured with B6MEFs infected with wild-type HSV-1(F) or YK513 (Us3-repair) was significantly greater than the amount of γ-IFN in the supernatants of IL-2 expanded NK cells co-cultured with B6MEFs infected with YK511 (Us3K220M) (Fig. 5A). In contrast, these differences in γ-IFN production were not observed with infected MHC-I^-/-^MEFs (Fig. 5B). These results indicated that Us3 kinase activity was required to make infected cells susceptible to NK-cell recognition and confirmed that Us3 kinase activity mediated down-regulation of cell surface expression of MHC-I in infected cells.

### Effect of HSV-1 Us3 kinase activity on HSV-1-specific CTL induction in vivo.

**Figure 6:**
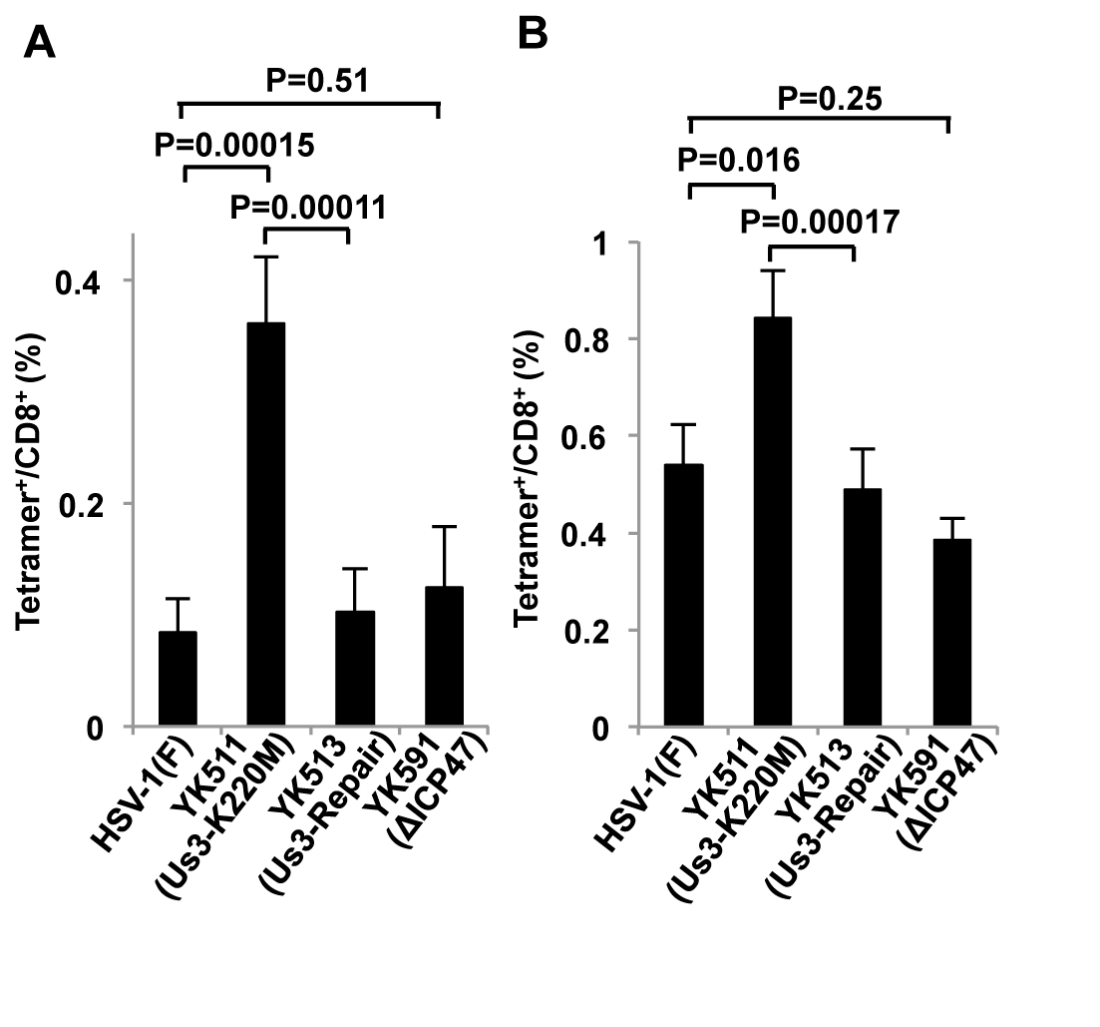
Effect of Us3 kinase activity on HSV-1-specific CTL induction in vivo. Six-week-old female C57BL/6J mice were mock-infected (n=21) or infected with 1 x 10^6^ PFU HSV-1(F) (n=21), YK511 (Us3-K220M) (n=21), YK513 (Us3-repair) (n=21) or YK591 (ΔICP47) (n=10)/footpad. At 4 d post-infection, spleen (A) and popliteal lymph node (B) cells were obtained and stained with MHC-I tetramers specific for the H-2K^b^-restricted HSV-1 gB immunodominant epitope (SSIEFARL). Cells were then stained with anti-CD8α and anti-CD3ε antibodies and analyzed by flow cytometry. The percentage of CD8^+^ and CD3^+^ cells from mock-infected mice that were positive for gB-specific MHC-I tetramer was subtracted from the percentage of CD8^+^ and CD3^+^ cells from mice infected with each virus that were also positive for gB-specific MHC-I tetramer. Each data point is the mean ± standard error.

To investigate the role of HSV-1 Us3 kinase activity in HSV-1-specific CTL induction in vivo, CD8^+^ T cells specific for the immunodominant gB_498-505_ epitope of HSV-1 [40] were measured in mice infected in a hind footpad with wild-type HSV-1(F), YK511 (Us3K220M), YK513 (Us3-repair) or YK591 (ΔICP47). As shown in Figure 6, YK511 (Us3K220M) elicited significantly more HSV-1-specific CTLs both in spleens and popliteal lymph nodes of mice than wild-type HSV-1(F), YK513 (Us3-repair) or YK591 (ΔICP47). These results indicated that Us3 kinase activity down-regulated HSV-1-specific CD8^+^ CTL induction in vivo.

### Effect of depletion of CD8^+^ T or NK cells in mice on replication of the Us3 kinase-dead mutant virus

**Figure 7:**
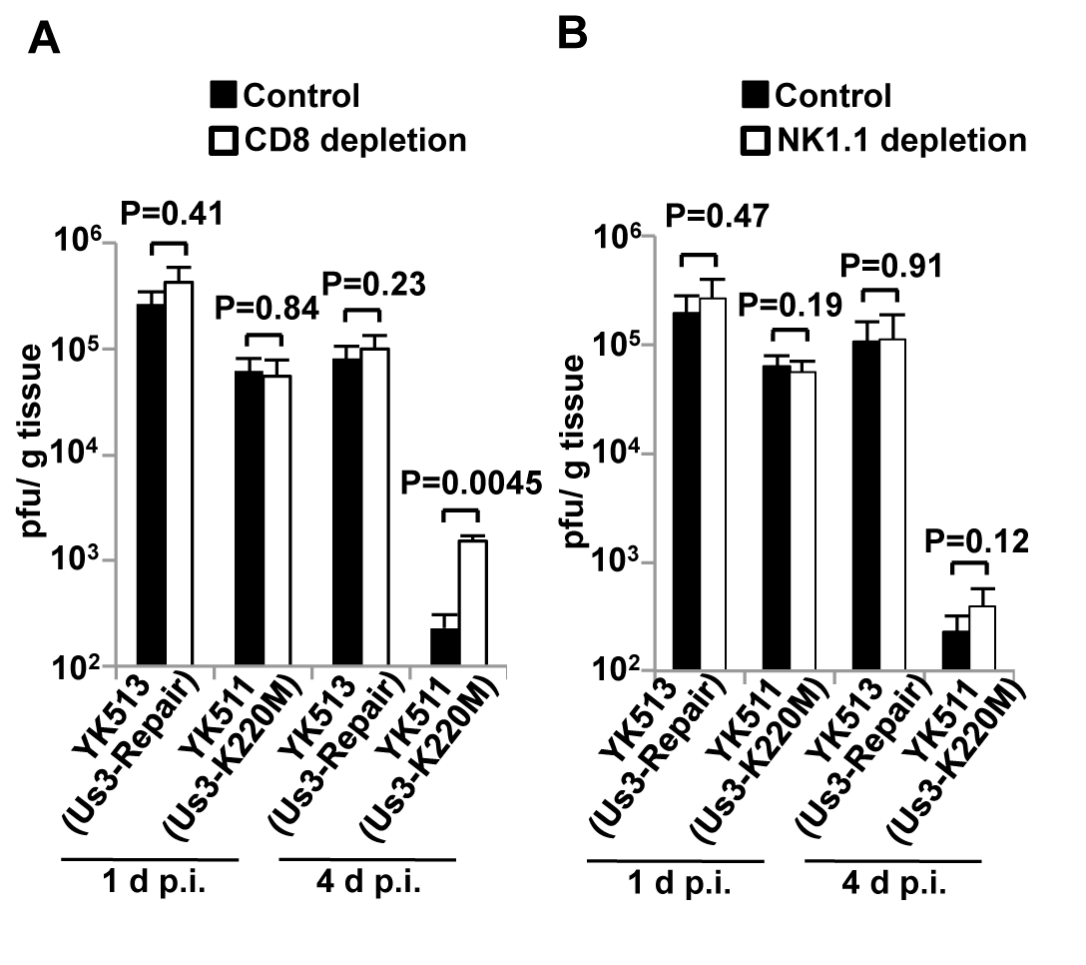
Effect of depletion of CD8^+^ T cells or NK cells on YK511 (Us3K220M) replication in vivo. Six 6-week-old female C57BL/6J mice were mock-depleted or depleted of CD8^+^ T cells (A) or NK1.1^+^ cells (B) and infected with 1 x 10^6^ PFU YK511 (Us3-K220M) or YK513 (Us3-repair)/footpad. At 1 and 4 d post-infection, virus titers in the footpads from the infected mice were determined by standard plaque assays on Vero cells. Each data point is the mean ± standard error of the PFU/gram/footpad.

As described above, Us3 kinase activity in infected cells appeared to have opposite effects on HSV-1-specific CTLs and NK cells co-cultured with infected cells with regard to elimination of the infected cells by immune system cells. Therefore, we examined which aspect of in vitro Us3 kinase activity was critical for viral replication in vivo. For this study, the hind footpads of mice were inoculated with YK511 (Us3K220M) or YK513 (Us3-repair), with or without CD8^+^ T cell- or NK cell-depletion, and virus titers in the footpads were assayed at 1 and 4 d post-infection. As shown in Figure 7B, depletion of NK cells had no significant effect on viral replication in mice infected with YK511 (Us3K220M) or YK513 (Us3-repair) at 1 and 4 d post-infection. Similarly, CD8^+^ T cell depletion had no effect on viral replication in the hind footpads of mice infected with YK511 (Us3K220M) or YK513 (Us3-repair) at 1 d post-infection, when HSV-1-specific CTLs were not induced [41]. However, at 4 d post-infection, when YK511 (Us3K220M) had induced more HSV-1-specific CTLs than YK513 (Us3-repair), as described above (Fig. 6), depletion of CD8^+^ T cells significantly (6-fold) increased replication of YK511 (Us3K220M) in the footpads, but had no effect on replication of YK513 (Us3-repair) (Fig. 7A). These results suggested that Us3-mediated evasion of CD8^+^ T cells might in part contribute to efficient viral replication in vivo.

## Discussion

Detailed studies of the molecular mechanisms by which alphaherpesvirus proteins act on the steps of the MHC-I antigen presentation pathway in vitro that have been reported to date [2] contrast with the lack of data on the in vivo functions of these proteins. In alphaherpesviruses, only HSV protein ICP47 has been reported to be involved in CD8^+^ T cell evasion in mice in vivo, as described above [14]. However, the reported effect of ICP47 on the MHC-I presentation pathway in vitro [8] is not the same in mouse cells [11,12], as we confirmed both in vitro and in vivo in this study. Therefore, the mechanism by which ICP47 functions in CD8^+^ T cell evasion in mice was unclear and indicated that a mechanism(s) other than inhibition of the MHC-I presentation pathway may be involved. We have shown here that inactivation of HSV-1 Us3 kinase activity significantly increased cell surface expression of MHC-I and HSV-1-specific antigen presentation in infected cells. These results were in agreement with the agonistic effect of the increased MHC-I cell surface expression on NK cell repression observed in this study. We also showed that inactivation of HSV-1 Us3 kinase activity induced significantly more HSV-1-specific CTLs in infected mice and that CD8^+^ T cell depletion significantly increased replication of the Us3 kinase-dead mutant virus in mice, although it had no effect on replication of the virus in which the Us3 mutation was repaired. Taken together, these experimental results indicated that Us3 kinase activity was required for efficient downregulation of cell surface expression of MHC-I and mediated evasion of HSV-1-specific CD8^+^ T cells and raised the possibility that this immune evasion might in part contribute to efficient viral replication in vivo. We note that the effect of Us3 on downregulation of MHC-I appeared to be indirect, based on the obaservations that overexpression of Us3 had no effect on cell surface expression of MHC-I and Us3 was not able to phosphorylate MHC-I in an in vitro kinase assay.

It has been reported that HSV-infected cells can inactivate CTL function and that Us3 is required for the CTL inactivation [42]. Based on this study, the increased activity of the HSV-1-specific CTL hybridoma clone against cells infected with the Us3 kinase-dead mutant virus compared to wild-type virus may not have been due to Us3-mediated inhibition of MHC-I cell surface expression, but to loss of the Us3 function that inactivated CTL. However, this seemed highly unlikely, because an HSV-1-specific CTL hybridoma clone incubated with MHC-I^-/-^MEFs infected by wild-type HSV-1 responded to wild-type HSV-1-infected B6MEFs at a level similar to the CTL hybridoma clone incubated with MHC-I^-/-^MEFs infected by the Us3 kinase-dead mutant virus (data not shown). Although an inhibitory effect of Us3 on the HSV-1-specific CTL hybridoma clone was not observed in this study, we cannot completely eliminate the possibility that Us3 inactivation of CTL was partly involved in the Us3-mediated inhibition of HSV-1-specific CD8^+^ T cells in mice found in this study.

Based on the observation that depletion of CD8^+^ T cells significantly increased replication of YK511 (Us3K220M) in the footpads of infected mice at 4 d post-infection, but had no effect on replication of YK513 (Us3-repair), we concluded that Us3 kinase activity resulted in increased viral replication in vivo by evasion of host CD8^+^ T cells. However, we noted that, at 4 d post-infection, viral replication of YK511 (Us3K220M) in the footpads of infected mice was remarkably reduced compared to replication of YK513 (Us3-repair). Although CD8^+^ T cell depletion significantly increased replication of YK511 (Us3K220M) in the footpads of mice at 4 d post-infection, replication of YK511 (Us3K220M) with CD8^+^ T cell depletion was still reduced compared to that of YK513 (Us3-repair). These results suggested that the reduction in YK511 (Us3K220M) replication in the footpads of mice at 4 d post-infection was not solely due to rapid loss of YK511 (Us3K220M) by CTLs. It has been reported that Us3 is a multi-functional viral protein kinase regulating a variety of cellular and viral activities in infected cells, including (i) blocking apoptosis [43–46], (ii) promoting nuclear egress of progeny nucleocapsids through the nuclear membrane (NM) [37,47,48], (iii) redistributing and phosphorylating NM-associated viral nuclear egress factors UL31 and UL34 and cellular factors lamin A/C and emerin [32,47,49–53], (iv) mediating phosphorylation of histone deacetylases (HDACs) and promoting gene expression by blocking histone deacetylation [54], (v) controlling infected cell morphology [29,45], (vi) down-regulating cell surface expression of viral envelope protein gB by promoting endocytosis of gB [30,31], (vii) stimulating mRNA translation by mimicking Akt and activating mTORC1 [55] and (viii) modulating host immune systems [20–22,56–58]. The defect in YK511 (Us3K220M) replication in the footpads of mice may have also been caused by inactivation of some of the Us3 functions that have been reported to promote viral replication in vivo [59,60].

Although we showed here that Us3 kinase activity resulted in NK cell recognition of infected cells in vitro, NK cell depletion had no effect on replication of wild-type or Us3 kinase-dead virus at the site of infection in mice following footpad inoculation. These observations were in agreement with a previous report [61] showing that NK cells do not significantly contribute to control of HSV-1 replication at the site of infection in mice following footpad inoculation. However, it has been reported that the effects of NK cells on HSV infection in mice are dependent on the route of viral inoculation, and NK cell depletion in mice following ocular, intraperitoneal and intravascular inoculation increased the susceptibility of mice to HSV infection [62]. Therefore, it would be interesting to compare the effects of depletion of NK and CD8^+^ T cells on HSV infection in mice inoculated by these routes.

Information on the mechanism(s) by which HSV evades the host cell immune response is important both for understanding viral strategies for survival in their hosts and for developing effective vaccines against HSV, which has not been clinically successful to date [1]. It has been reported that UL41 is involved in inhibition of maturation of dendritic cells, key cells in stimulating and directing the development of adaptive immune responses [63], and that disruption of UL41 increases the immunogenicity and protective capacity of a potential, replication-incompetent HSV vaccine [64]. The present study, showing that inactivation of Us3 kinase activity increased HSV-1-specific CTL induction in mice, suggested that disruption of Us3 may be another method for increasing the immunogenicity and protective capacity of potential HSV vaccines. Furthermore, it has been reported that Us3 mutant viruses exhibited remarkably reduced virulence and capacity for latent infection in mice [59,60]. These observations raised the possibility that HSV-1 Us3 mutant viruses could be potential candidates for novel, safe, live-attenuated, vaccine platforms against HSV-1 infection.

## Supporting Information

Figure S1 **Verification of CD8^+^ (A and C) and NK1.1^+^ (B and >D) cell depletion in C57BL/6J mice.** Six-week-old C57BL/6J mice were injected intraperitoneally with PBS, or 200 µg anti-CD8α or NK1.1 antibody. Cells from spleen and popliteal lymph nodes were stained with anti-CD8α or anti-NK 1.1 antibody and analyzed by flow cytometry 2 d after antibody administration. The results of first (A and B) and second (C and D) experiment are shown as log-log dot plots.
(TIF)

Figure S2 **(A) Effect of Us3 expression on cell surface expression of MHC-I in 293T cells.** 293T cells were co-transfected with 1.6 µg of pEGFP-C1 in combination of 1.6 µg pFLAG-Us3 or pFLAG-CMV2. At 48 h after transfection, cell surface expression of MHC-I in transfected cells were analyzed by flow cytometry. (**B**) **Us3 does not directly phosphorylate MHC-I in vitro**. Puriﬁed MBP-gB (lane 1), MBP-gB-T887A (lane 2), MBP-MHC-I (lanes 3 and 5) and MBP-MHC-I-SS336/337AA (lanes 4 and 6) were incubated in kinase buffer containing [γ-^32^P] ATP and puriﬁed GST-Us3 (lanes 1 to 4) or protein kinase A (PKA) (lanes 5 and 6), separated on a denaturing gel, and stained with CBB (upper panel). An autoradiograph of the gel in upper panel is shown in the lower panel.
(TIF)

Figure S3 **Effect of Us3 kinase activity and vhs enzymatic activity on viral growth in MRC-5 and B6MEF cells.** MRC-5 (A and B) and B6MEF cells (C and D) were infected at an MOI of 3 (A and C) or 0.01 (B and D) with each of the indicated wild-type and recombinant viruses. Total virus from the cell culture supernatants and the infected cells was harvested at the indicated times and assayed on Vero cells.
(TIF)

Figure S4 **Effect of Us3 kinase activity and vhs enzymatic activity on expression of β-actin mRNA in infected cells.** MRC-5 (A) and B6MEF cells (B) were mock-infected or infected with each of the indicated wild-type and recombinant viruses at an MOI of 3, harveted at 18 h post-infection and the amount of β-actin mRNA was analyzed by quantitative RT-PCR. Each bar is the mean ± standard error of data from three independent experiments. The mean value for each of the indicated viruses was calculated relative to that for the corresponding mock-infected cells, which was normalized to 100.
(TIF)

Figure S5 **Effect of vhs enzymatic activity on cell surface and total expression of MHC-I in HSV-1-infected MRC-5 cells.** (A) Surface expression of MHC-I in MRC-5 cells infected with HSV-1(F), YK511 (Us3-K220M), YK478 (UL41-D213N) or YK479 (UL41D213N-repair) at an MOI of 3 for 18 h and analyzed and quantitated as described in (Figure 2B). Each data point is the mean ± standard error of triplicate samples, and is representative of three independent experiments. (B) Total accumulation of MHC-I in MRC-5 cells mock-infected ot infected with HSV-1(F), YK511 (Us3-K220M), YK478 (UL41D213N) or YK479 (UL41D213N-repair) at an MOI of 3 for 18 h and analyzed and quantitated as described in (Figure 2B). The data were calculated relative to mock-infected cells, which was normalized to 100. Each data point is the mean ± standard error of triplicate samples, and is representative of three independent experiments.
(TIF)

Figure S6 **Effect of vhs enzymatic activity on cell surface and total expression of MHC-I (H-2Kb and H-2Db) in HSV-1-infected B6MEFs.** (A and C) Surface expression of H-2Kb (A) and H-2Db (C) in B6MEFs infected with HSV-1(F), YK511 (Us3-K220M), YK478 (UL41D213N) or YK479 (UL41D213N-repiar) at an MOI of 3 for 18 h and analyzed and quantitated as described in (Figure 2B). Each data point is the mean ± standard error of triplicate samples, and is representative of three independent experiments. The data were calculated relative to mock-infected cells, which was normalized to 100. (B and D) Total accumulation of H-2Kb (B) and H-2Db (D) in B6MEFs mock-infected or infected with HSV-1(F), YK511 (Us3-K220M), YK478 (UL41D213N) or YK479 (UL41D213N-repair) at an MOI of 3 for 18 h and analyzed and quantitated as described in (Figure 2B). The data were calculated relative to mock-infected cells, which was normalized to 100. Each data point is the mean ± standard error of triplicate samples, and is representative of three independent experiments.
(TIF)

Figure S7 **Effect of vhs enzymatic activity on HSV-1-specific antigen presentation.** B6MEFs were infected with wild-type HSV-1(F), YK511 (Us3-K220M), YK478 (UL41-D213N) or YK479 (UL41-D213N-repair) at an MOI of 1 for 12 h and then co-cultured for an additional 12 h with *lacZ*-inducible CTL hybridoma cells recognizing HSV-1 gB (HSV-2.3.2E2), followed by β-galactosidase assays. Each data point is the mean ± standard error of triplicate samples, and is representative of three independent experiments.
(TIF)
