# Supplementary figures and images for: Us3 Kinase Encoded by Herpes Simplex Virus 1 Mediates Downregulation of Cell Surface Major Histocompatibility Complex Class I and Evasion of CD8+ T Cells
Source: PLoS One. 2013 Aug 12;8(8):e72050. doi: 10.1371/journal.pone.0072050 (PMC3741198; doi:10.1371/journal.pone.0072050)

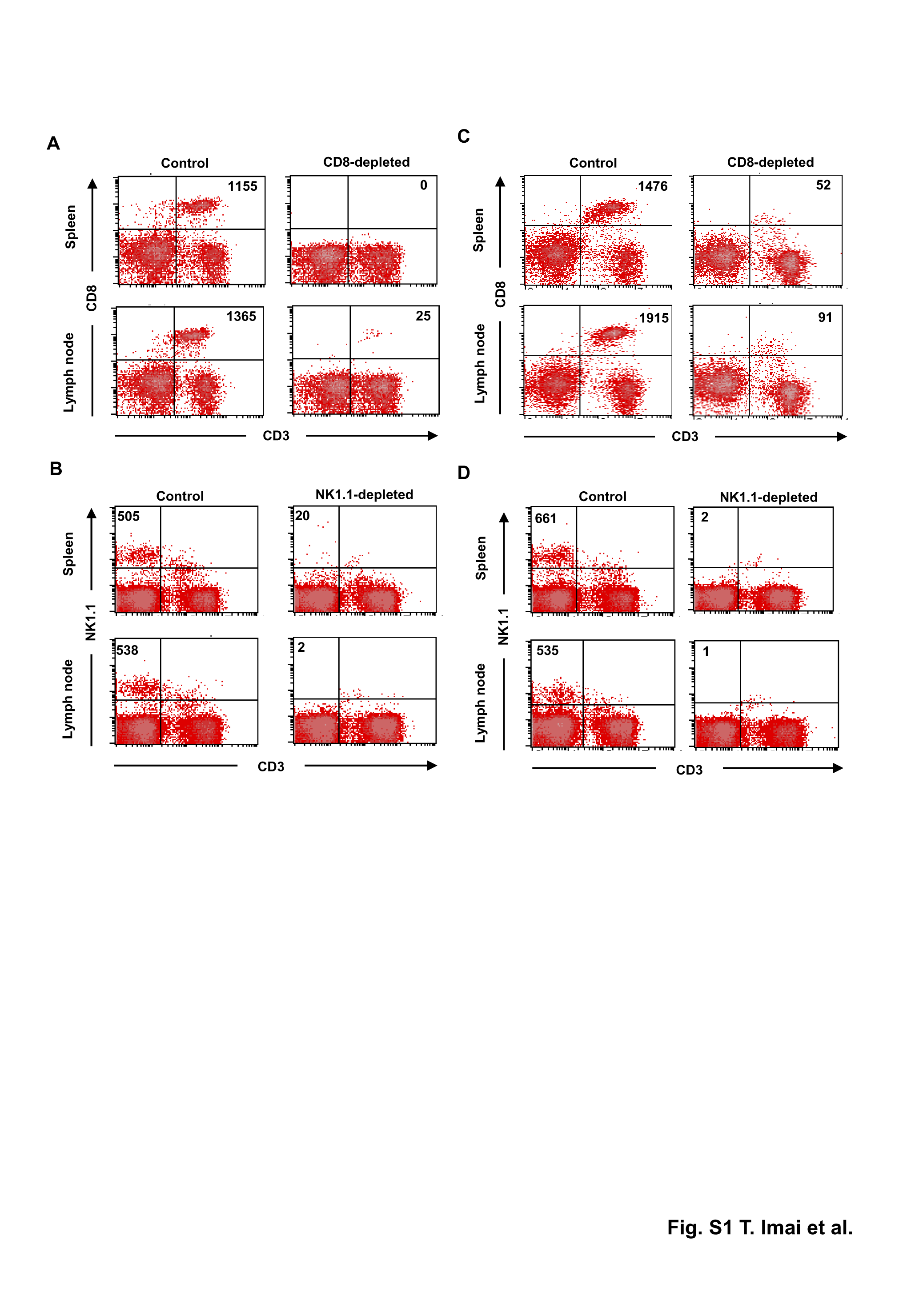

Supplement: Figure S1 — Verification of CD8+ (A and C) and NK1.1+ (B and >D) cell depletion in C57BL/6J mice. Six-week-old C57BL/6J mice were injected intraperitoneally with PBS, or 200 µg anti-CD8α or NK1.1 antibody. Cells from spleen and popliteal lymph nodes were stained with anti-CD8α or anti-NK 1.1 antibody and analyzed by flow cytometry 2 d after antibody administration. The results of first (A and B) and second (C and D) experiment are shown as log-log dot plots. (TIF) [file pone.0072050.s001.tif]

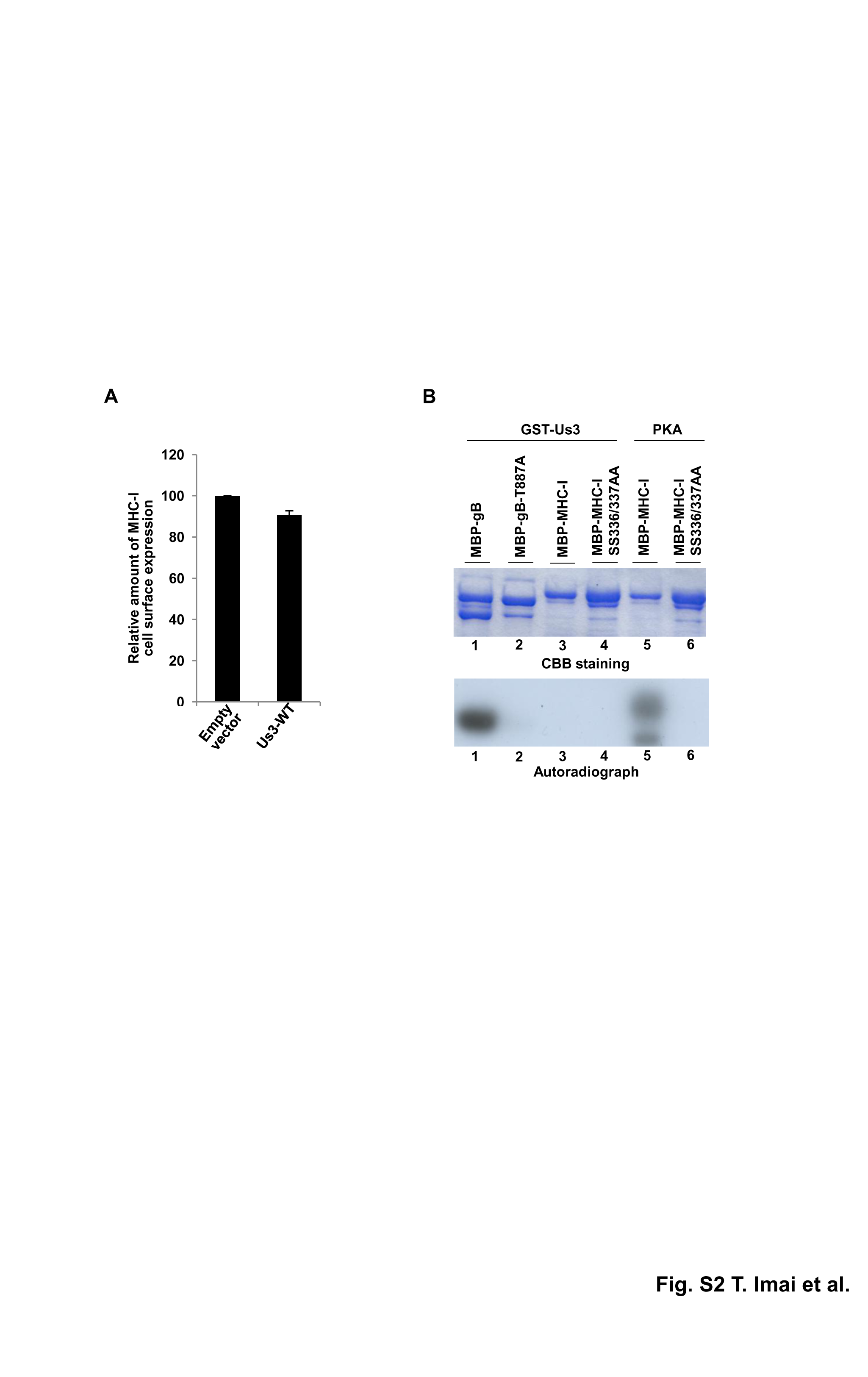

Supplement: Figure S2 — (A) Effect of Us3 expression on cell surface expression of MHC-I in 293T cells. 293T cells were co-transfected with 1.6 µg of pEGFP-C1 in combination of 1.6 µg pFLAG-Us3 or pFLAG-CMV2. At 48 h after transfection, cell surface expression of MHC-I in transfected cells were analyzed by flow cytometry. (B) Us3 does not directly phosphorylate MHC-I in vitro. Puriﬁed MBP-gB (lane 1), MBP-gB-T887A (lane 2), MBP-MHC-I (lanes 3 and 5) and MBP-MHC-I-SS336/337AA (lanes 4 and 6) were incubated in kinase buffer containing [γ-32P] ATP and puriﬁed GST-Us3 (lanes 1 to 4) or protein kinase A (PKA) (lanes 5 and 6), separated on a denaturing gel, and stained with CBB (upper panel). An autoradiograph of the gel in upper panel is shown in the lower panel. (TIF) [file pone.0072050.s002.tif]

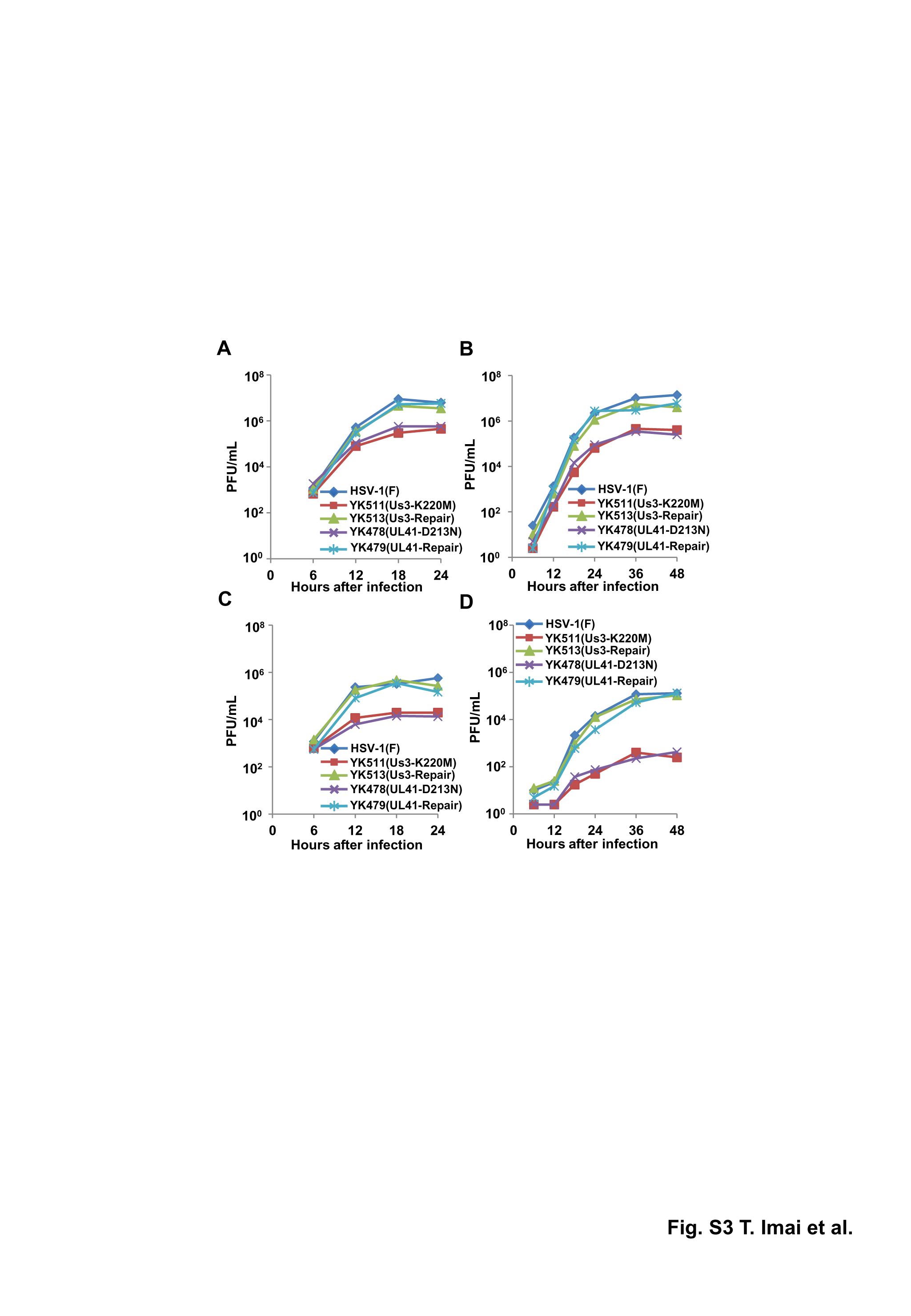

Supplement: Figure S3 — Effect of Us3 kinase activity and vhs enzymatic activity on viral growth in MRC-5 and B6MEF cells. MRC-5 (A and B) and B6MEF cells (C and D) were infected at an MOI of 3 (A and C) or 0.01 (B and D) with each of the indicated wild-type and recombinant viruses. Total virus from the cell culture supernatants and the infected cells was harvested at the indicated times and assayed on Vero cells. (TIF) [file pone.0072050.s003.tif]

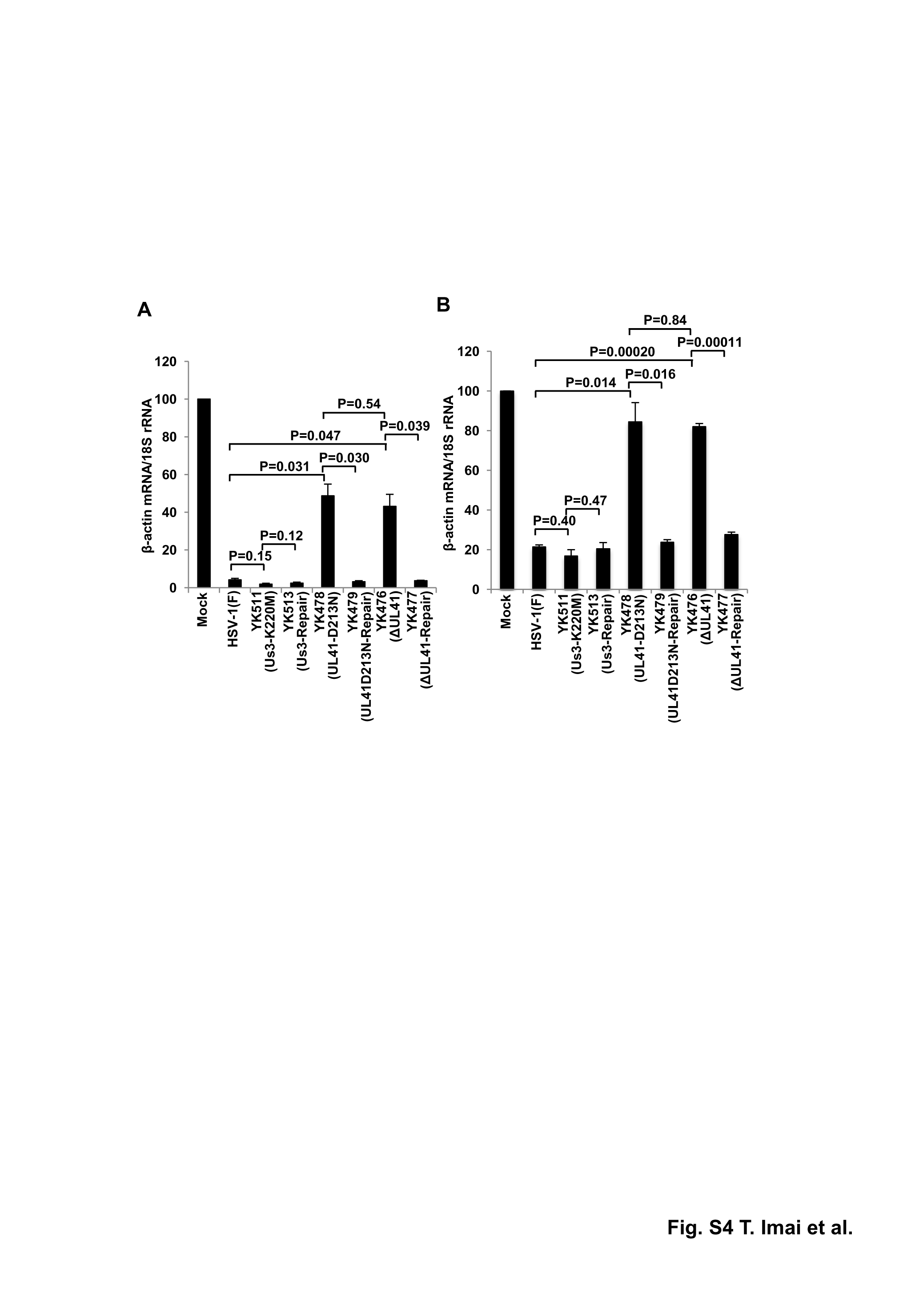

Supplement: Figure S4 — Effect of Us3 kinase activity and vhs enzymatic activity on expression of β-actin mRNA in infected cells. MRC-5 (A) and B6MEF cells (B) were mock-infected or infected with each of the indicated wild-type and recombinant viruses at an MOI of 3, harveted at 18 h post-infection and the amount of β-actin mRNA was analyzed by quantitative RT-PCR. Each bar is the mean ± standard error of data from three independent experiments. The mean value for each of the indicated viruses was calculated relative to that for the corresponding mock-infected cells, which was normalized to 100. (TIF) [file pone.0072050.s004.tif]

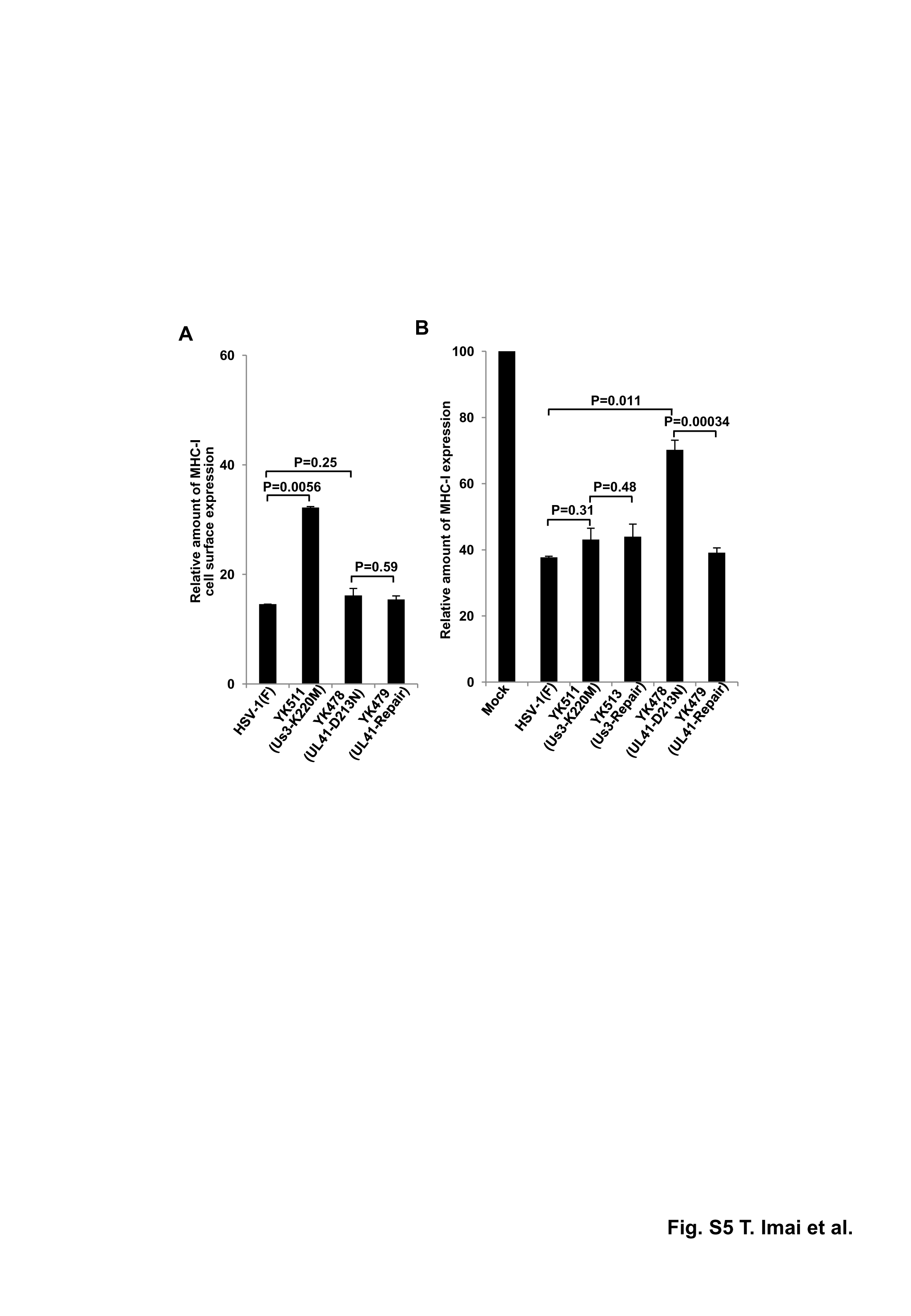

Supplement: Figure S5 — Effect of vhs enzymatic activity on cell surface and total expression of MHC-I in HSV-1-infected MRC-5 cells. (A) Surface expression of MHC-I in MRC-5 cells infected with HSV-1(F), YK511 (Us3-K220M), YK478 (UL41-D213N) or YK479 (UL41D213N-repair) at an MOI of 3 for 18 h and analyzed and quantitated as described in (Figure 2B). Each data point is the mean ± standard error of triplicate samples, and is representative of three independent experiments. (B) Total accumulation of MHC-I in MRC-5 cells mock-infected ot infected with HSV-1(F), YK511 (Us3-K220M), YK478 (UL41D213N) or YK479 (UL41D213N-repair) at an MOI of 3 for 18 h and analyzed and quantitated as described in (Figure 2B). The data were calculated relative to mock-infected cells, which was normalized to 100. Each data point is the mean ± standard error of triplicate samples, and is representative of three independent experiments. (TIF) [file pone.0072050.s005.tif]

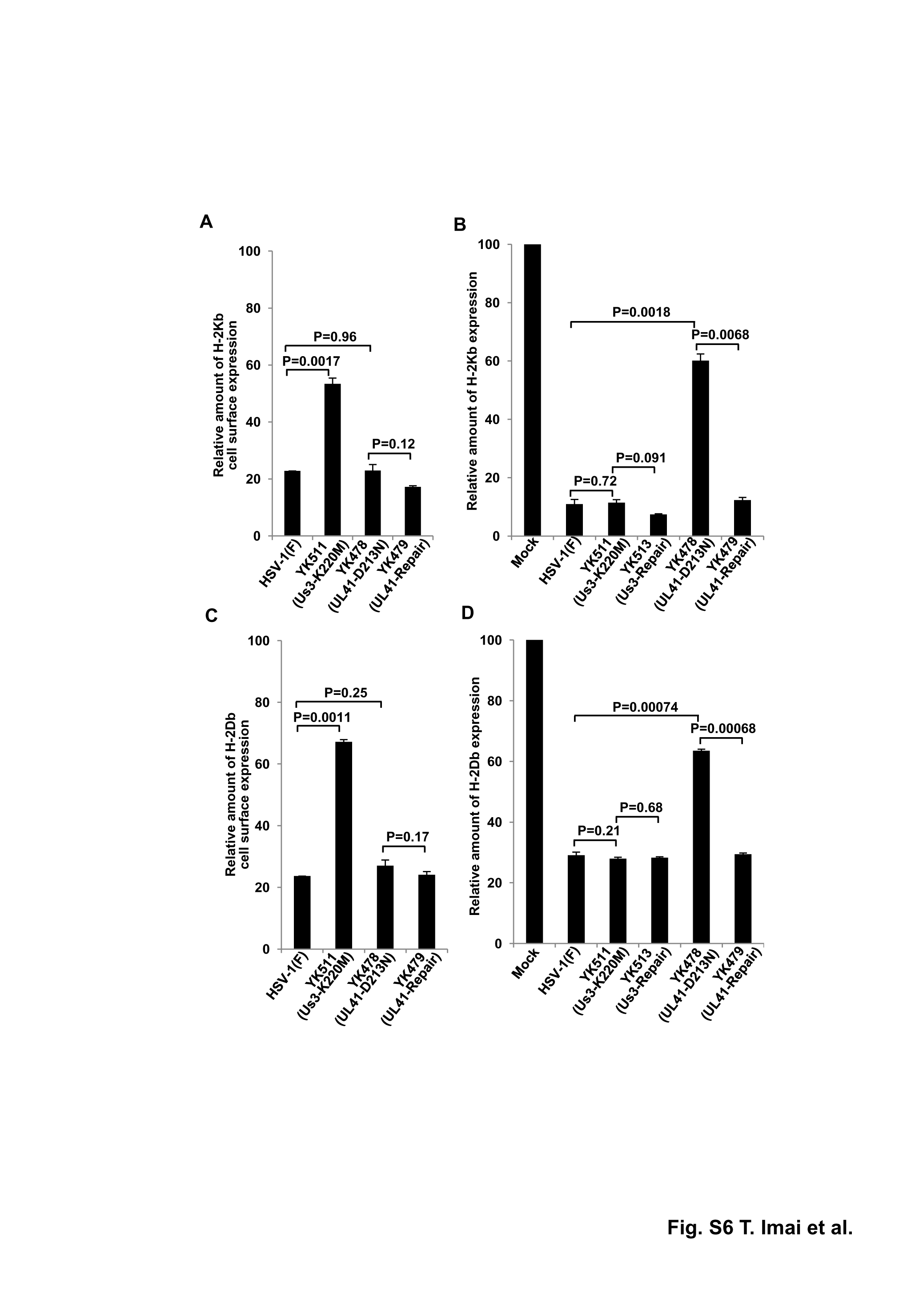

Supplement: Figure S6 — Effect of vhs enzymatic activity on cell surface and total expression of MHC-I (H-2Kb and H-2Db) in HSV-1-infected B6MEFs. (A and C) Surface expression of H-2Kb (A) and H-2Db (C) in B6MEFs infected with HSV-1(F), YK511 (Us3-K220M), YK478 (UL41D213N) or YK479 (UL41D213N-repiar) at an MOI of 3 for 18 h and analyzed and quantitated as described in (Figure 2B). Each data point is the mean ± standard error of triplicate samples, and is representative of three independent experiments. The data were calculated relative to mock-infected cells, which was normalized to 100. (B and D) Total accumulation of H-2Kb (B) and H-2Db (D) in B6MEFs mock-infected or infected with HSV-1(F), YK511 (Us3-K220M), YK478 (UL41D213N) or YK479 (UL41D213N-repair) at an MOI of 3 for 18 h and analyzed and quantitated as described in (Figure 2B). The data were calculated relative to mock-infected cells, which was normalized to 100. Each data point is the mean ± standard error of triplicate samples, and is representative of three independent experiments. (TIF) [file pone.0072050.s006.tif]

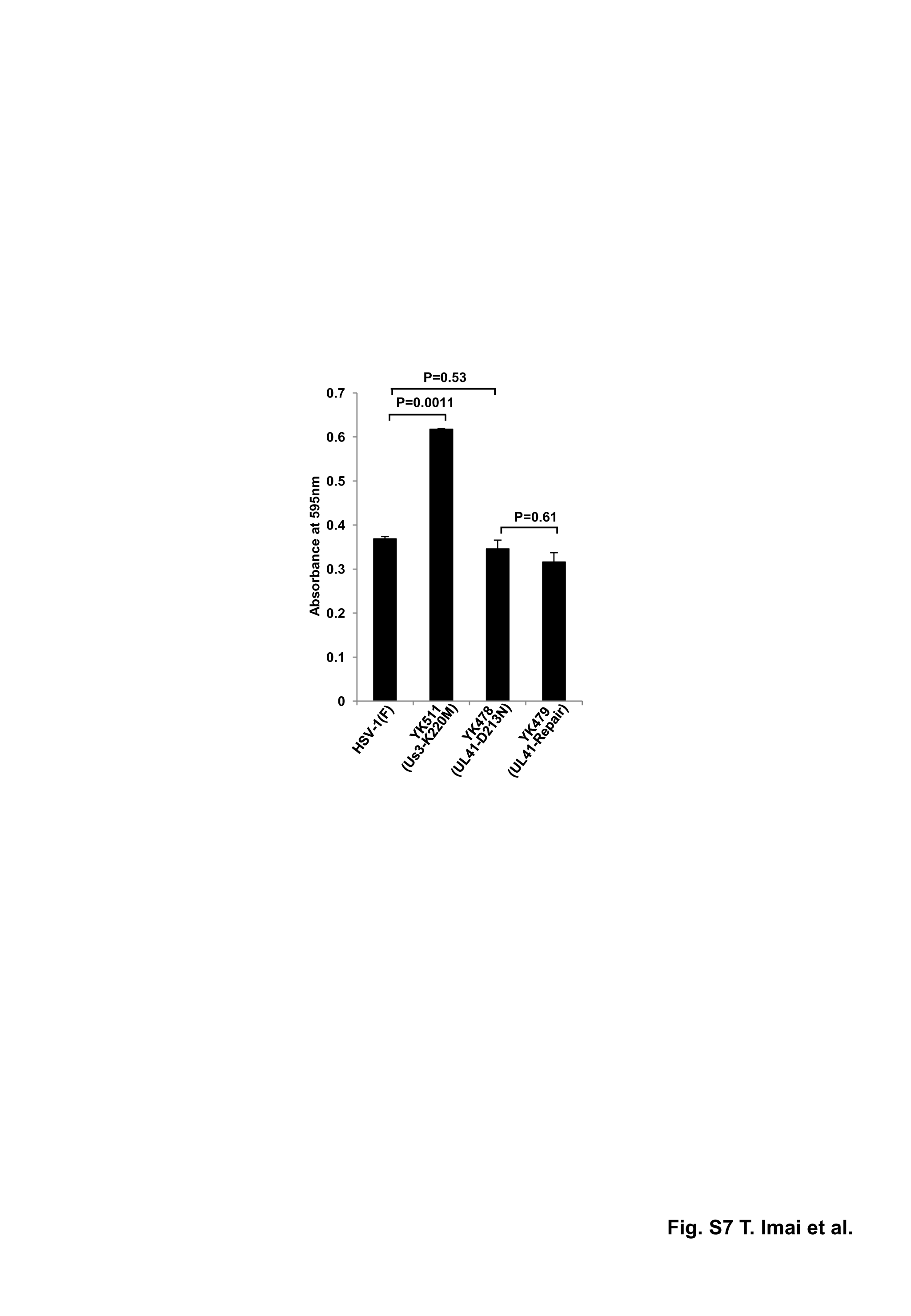

Supplement: Figure S7 — Effect of vhs enzymatic activity on HSV-1-specific antigen presentation. B6MEFs were infected with wild-type HSV-1(F), YK511 (Us3-K220M), YK478 (UL41-D213N) or YK479 (UL41-D213N-repair) at an MOI of 1 for 12 h and then co-cultured for an additional 12 h with lacZ-inducible CTL hybridoma cells recognizing HSV-1 gB (HSV-2.3.2E2), followed by β-galactosidase assays. Each data point is the mean ± standard error of triplicate samples, and is representative of three independent experiments. (TIF) [file pone.0072050.s007.tif]
